# Hand-grip strength is a simple and effective outcome predictor in esophageal cancer following esophagectomy with reconstruction: a prospective study

**DOI:** 10.1186/1749-8090-6-98

**Published:** 2011-08-15

**Authors:** Chih-Hao Chen, Ho-Chang, Yi-Zhen Huang, Tzu-Ti Hung

**Affiliations:** 1 Department of Thoracic Surgery, Mackay Memorial Hospital, Taipei City, Taiwan; 2 Mackay Medicine, Nursing and Management College, Taipei City, Taiwan; 3 Graduate Institute of Mechanical and Electrical Engineering and Graduate Institute of Manufacturing Technology, National Taipei University of Technology, Taipei City, Taiwan

## Abstract

**Background:**

Surgery for esophageal cancer usually carries considerable complication and mortality rate. Adequate preoperative evaluation is mandatory to decrease complication rate. Hand-grip strength is a useful measure to assess the extent of aging, nutrition and patient's overall condition. Because preoperative nutrition state and physiologic aging process play important roles in postoperative recovery, we would like to know if hand-grip strength is an adequate tool for such evaluation.

**Material and methods:**

From January 1st, 2007 to December 31, 2008, there was 68 cases underwent esophagectomy with reconstruction due to esophageal cancer in our hospital. After excluding 7 patients of incomplete data and loss of follow-up, there were 61 patients included in the study.

**Results:**

There were 54 men and 7 women. The mean age is 60.7. Most of patients had squamous cell carcinoma. Patient with weak hand-grip strength prior to operation had exceedingly high rates of complication and mortality within 6 months after operation. Compared to other risk factors, low grip strength has highest relative risks for both mortality and morbidity.

**Conclusion:**

Because test for hand-grip strength is cheap, not time-consuming and has high predictive value, it may be included in routine preoperative evaluation.

## Background

Patients with esophageal cancer often present with dysphagia and generalized weakness. Resection of the esophageal tumor with concomitant reconstruction, with either stomach or colon, is the procedure of choice. However, such procedure still carries considerable complication rates. For advanced disease, life expectance is often less than 12 months. Hence, adequate preoperative survey is necessary for all potential surgical candidates because complication and mortality would definitely occurred in a certain portion of patients. One of the important values of preoperative evaluation is to define those with high risks for morbidity and mortality. In addition to routine cancer survey, we often would like to evaluate patient's cardiac function or lung function as well as laboratory examinations. An ideal evaluation tool may be cheap, easy to interpret, not time consuming, not space-occupying, and effective. Hand-grip strength is a proper predictor of immune system, nutrition, aging process, bone density, overall body strength, especially in old age group [1]. The methods of such test is quite simple, we therefore want to see if such test has any role to predict patient's outcome after esophagectomy with reconstruction. The end-points are ICU stay, hospital stay, complication rate, days to start oral intake, surgical mortality rate, andmortality rate within 6 months after operation.

## Material and methods

From January 1^st^, 2007 to December 31^th^, 2008, there were 68 patients included in the study. This is a prospective study. The study was approved by the institution review board of Mackay Memorial Hospital. 7 patients were excluded due to loss of follow-up, could not adequately perform the tests due to stroke or due to incomplete data, Patients with other cancer history were also excluded. There were 61 patients have complete records for analysis. Patients were tested at least 3 times several days prior to operation. Low hand-grip strength here was defined as grip strength lower than 25 kg in the dominant hand. Other laboratory data, history of other co-morbidities and risk factors were also recorded for risk analysis. The standard position for testing hand-grip strength is standing position with upper limb relaxed down to the sides of the body and palm towards the torso. The elbow is extended without any flexion. The handedness is also recorded for comparison. Co-morbidities included diabetes, poor renal function, hypertension, ischemic heart disease, liver cirrhosis or other disease considered to have great influence on patients' outcome. Complications included postoperative acute respiratory failure, anastomotic leakage, wound infection, early esophageal stricture requiring endoscopic dilatation, and pleural effusion requiring tube drainage. Surgical mortality was defined as either patients died within 30 days after operation or in-hospital death without discharge. Mortality was defined to any patients died less than 6 months after operation during follow-up. Pathology stage was based on resected specimens. Early stage was defined as patients having stage 1 and stage 2. Advanced stage was defined as patients having stage 3 and 4. All patients included were followed for at least 6 months in the outpatient department.

SPSS (version 13.0) was used to help analyze the correlation of each risk factors with morbidity, mortality and hospital stay. Chi square test, Student t-test and Pearson correlation test were used to compare the influences of each factor. Receiver operating curve analysis was used to determine the most appropriate cut-off value of the tests. Regression analysis was used to evaluate the influence of each factor on outcome.

## Results

There were 54 men and 7 women with mean age of 60.7 years.(range: 34 - 83 years). All patients had undergone esophagectomy with reconstruction by a gastric tube through either transhiatal or transthoracic approach. Transthoracic esophagectomy with reconstruction was performed in 52 patients and transhiatal esophagectomy with reconstruction was performed in 9 patients. Summary of the surgical approach and other variables is shown in table 1. Only five patients underwent thoracoscopic esophagectomy with reconstruction in the group of transthoracic approach. Fifty-seven patients had squamous cell carcinoma and the remaining 4 patients had adenocarcinoma. The locations included 6 patients in the upper third, 31 patients in the middle third and 14 in the lower third. All underwent surgery for cure intent, including en-bloc resection of the tumor, esophagus and radical nodal dissection. 93.4%(57 out of 61 patients) had right handedness. For risk factors, anemia was found to be in 3 patients, hypoalbulinemia in 10 patients, chronic renal insufficiency in 8, diabetes in 11, abnormally elevated MCV (mean cellular volume of red cells greater than 100 fL) in 22, and presence of weight loss in 29. Twenty-three patients had at least one major comorbidity. The postoperative routine included observation in intensive care unit for one to three days after operation depending on patient's condition, removing endotracheal tube one day after operation, removing nasogastric tube on day 8 and began oral intake 10 to 12 days after operation when recovery was uneventful.

**Table 1:** Surgical Approach of patients with operable esophageal cancer

	TTE	THE	p value
NO	52	9	
M/F	48/4	6/3	0.1
Age (year)	60.6	61	0.92
HGS(kg)	29.1	26.5	0.33
HS (day)	25.4	22.2	0.46
ICU stay(day)	7.26	4.7	0.21
Start Oral Intake(day)	15.4	13.1	0.6
6-month mortality	6	2	0.5
Morbidity	22	8	0.06

TTE: transthoracic esophagectomy with reconstruction (including thoracoscopic and thoracotomy); THE: transhiatal esophagectomy with reconstruction; NO: patient number; M: male; F: female; HGS: hand-grip strength; HS: hospital stay; ICU: intensive care unit.

Respiratory failure requiring re-intubation was found in 12 patients, pneumonia in 7 patients, pleural effusion requiring tube drainage in 5, anastomotic leak in 3 and other conditions in 4 patients. Surgical mortality was found in 6 patients. 8 patients died within 6 months after radical operation. The mean duration to start regular oral intake is 15.1 days. For patients with tumor in the upper third of the esophagus, 2 out of 6 patients had complication but none died of disease within 6 months. For tumor in the middle third, 3 out of 31 patients died and 15 patients had complications. For tumor in the lower third, 5 patients died within 6 months and 12 had complications.

**Figure 1:**
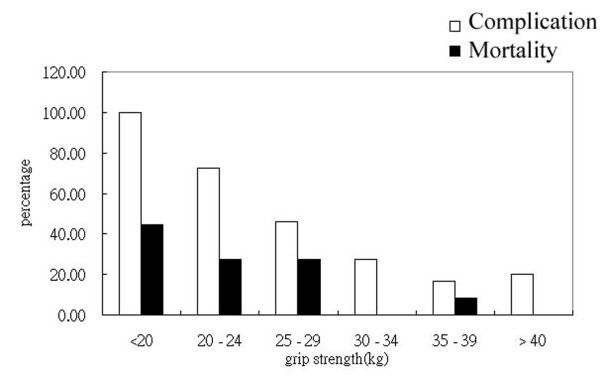
**The rates of complication and mortality in each group of hand-grip strength**. It showed marked increased rates of complication and mortality when the strength of grip decreased. (p < 0.0001 and p = 0.003 respectively)

**Figure 2:**
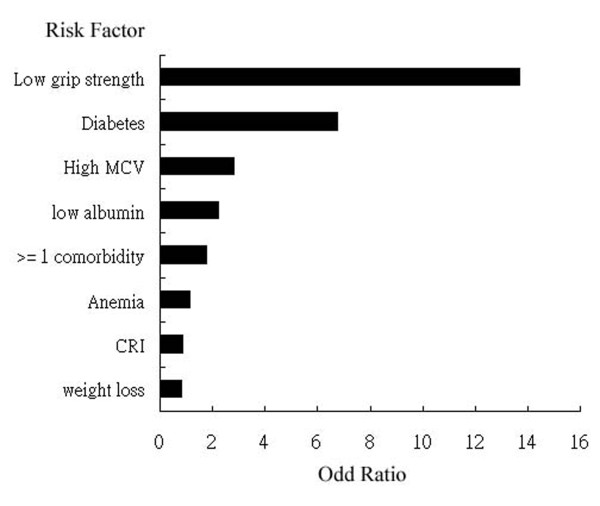
**The odd ratio and relative risk of each risk factors for complication rate**. Both weak hand-grip strength and diabetes were associated with more likelihood of complication. (p value less than 0.05) Abnormally elevated MCV has borderline significance.

Figure 1 showed highest rates of complication and mortality when hand-grip strength was less than 20 kg and lowest when hand-grip strength greater than 40 kg. The extent of correlation between hand-grip strength with complication rate and mortality rate reached statistical significance (*p *value less than 0.001 and 0.020 respectively) The correlation coefficients are -0.546 for complication rate and -0.369 for mortality rate. The correlation coefficients between age and mortality as well as morbidity were -0.052 and 0.122 respectively and not reached statistical significance. Table 2 showed the likelihood of complication and 6-month mortality rate decreased along with better hand-grip strength. When compare to the presence of other risk factors, low hand-grip strength still carries highest rate of complication, followed by patients with diabetes.(Figure 2) Abnormally elevated MCV has borderline significance.(p = 0.059)

**Table 2:** The clinical demographics and outcome in patients with weak(< 25 kg) and normal hand-grip strength

		Weak HGS	Normal HGS	p value
N.O.		20	41	
Sex(M/F)		17/3	37/4	0.742
HGS(mean, kg)		19.325	33.34	< 0.0001
Age(mean, years)		65.1	58.5	0.01
weight loss		10	19	0.57
stage	Early(I, II)	12	23	0.625
	Advanced(III, IV)	8	18	
ICU stay(day)		12.75	4.05	0.003
Hospital stay(day)		32.3	21.4	0.003
Start oral intake(day)		22.1	12.02	0.001
Complication		17	12	< 0.0001
Mortality		7	2	0.016

**Figure 3:**
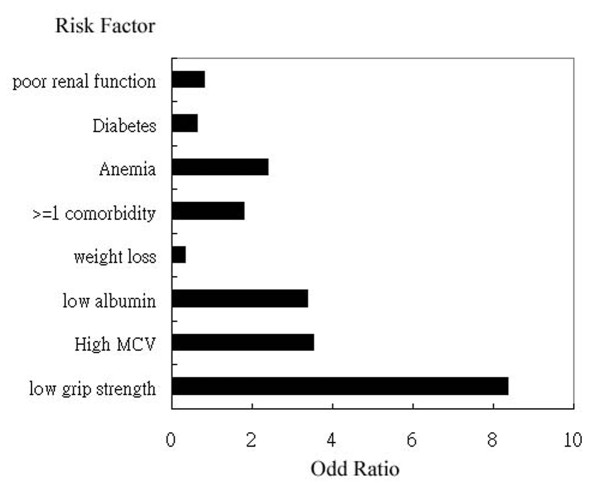
**The odd ratio and relative risks of each risk factor for mortality rate**. Only weak hand-grip strength has significantly associated with more mortality rates. Other risk factors were not associated with mortality rate.

HGS: hand-grip strength; M, male; F, female; ICU, intensive care unit;

Factors thought to be significant to predict surgical mortality included weak hand-grip strength, chronic renal insufficiency and presence of at least one co-morbidity. *P *values are 0.034, 0.038 and 0.036, respectively. When the duration extended to 6-month, the condition is somewhat different. Only weak hand-grip strength correlated significantly with mortality rate. The *p *value is 0.016.(Figure 3) Other risk factors have no significance to predict 6-month mortality. For patients with low hand-grip strength, the mean duration to start regular oral intake is significantly longer than those with stronger hands.(22 days vs 12 days, p = 0.001) The hospital stay is also significantly longer in patients with weak grip strength. (32.3 days vs 21.4 days, p = 0.005) In the group of advanced age (more than 65 years old), the hospital stay was not longer than young patients. As for the influence of stage of esophageal cancer, the average hand-grip strength was similar in patients having early and advanced esophageal cancer.(p value is 0.961) Advanced esophageal cancer itself did not contribute more likelihood of mortality and complication within 6 months after operation.(p value is 0.229 and 0.177, respectively)

**Figure 4:**
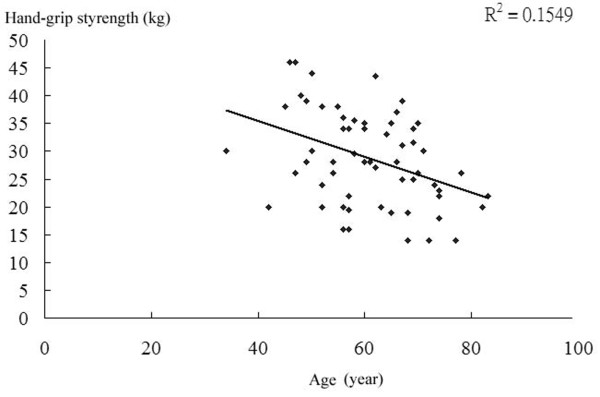
**The figure showed that hand-grip strength does declined with age**.

**Figure 5:**
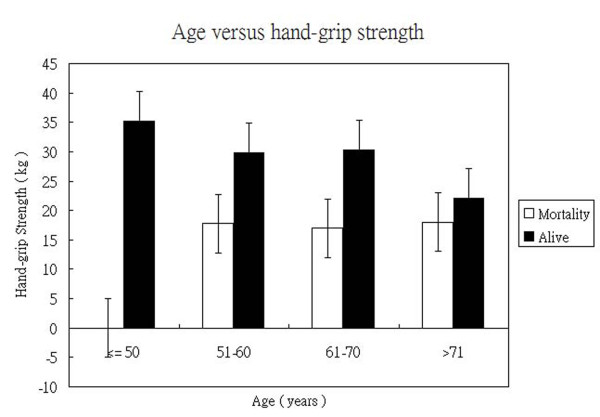
**The hand-grip strength in mortality and survived patients was stratified according to age**. The mean hand-grip strength in mortality patients is significantly lower than survived patients in the range from 51 to 60 years and 61 to 70 years.(p value was 0.024 and 0.014 respectively) The difference in patients older than 71 years old was not significant.

Because the strength of hand-grip declined with age (Figure 4), we tried to stratify all patient according to age and then assess if hand-grip strength still contribute to more likelihood of mortality. Figure 5 showed when patients younger than 50 years, there was no mortality. In patients aged 51 to 60 years, the mean hand-grip strength is 17.75 kg, significantly lower than 29.89 kg in survived group.(p value: 0.024) In patients aged 61 to 70 years, the mean hand-grip strength is 17 kg, significantly lower then 30.3 kg in survived patients.(p value: 0.014). In patients aged more 71 years, the mean hand-grip strength is 18 kg, only slightly lower then 22.13 kg in survived group.(p value: 0.33) The facts described that weak strength is an adequate indicator of poor outcome in patients aged from 51 to 70 years but the predictive value in patients more then 71 years old is not confirmed. The strength of non-dominant hand has no impact on patients' outcome in the analysis.

In brief, patients who have weak hand-grip strength prior to operation tends to stay longer in the hospital, need longer time to start oral diet and may have more risks for occurrence of complication and mortality.

## Discussion

Hand-grip strength is a useful marker to assess patient's physiologic status [2]. It correlates well to patient's overall muscle strength, bone density, nutrition status, and frailty, even better than chronological age [3]. The reason to test muscle strength to determine the aging process is that muscle-specific disease is quite rare. That means muscle strength declined slowly independent of other common disease, such as heart disease, lung disease or gastrointestinal disturbance [4]. This independence helps this marker as a life-long tool to evaluate frailty. Although we all know the number of age itself, that is chronologic age, could not represent patient's true physiologic state, we do not use any better and reliable measurements to take the place of chronological age in our routine clinical setting. Hand-grip strength can be a proper and useful measurement here. Other evaluations, for example cardiac ultrasound or lung function test, often require expensive facility and well-trained personnel. Interpretation of the results is also complicated and required experience. On the contrary, performing the test of hand-grip strength costs less than one minute for both hands and the instructions are simple. Personnel who perform the test need essentially minimal training but keep the patient in adequate position and then read the number. A simple dynamometer can be used for at least one to two thousand times under adequate calibration. It can be a simple, reliable and cheap measurement.

**Figure 6:**
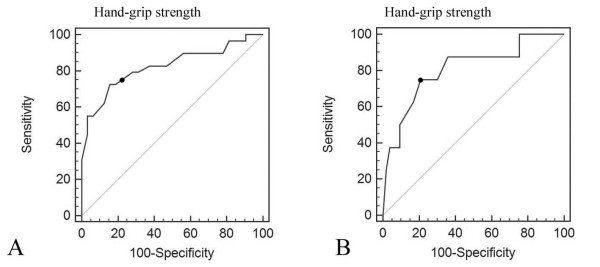
**Receiver operating characteristic curve showed the most appropriate cut-off value is 25 kg for morbidity(A) and 22 kg for mortality(B)**.

The reason we define hand-grip strength less than 25 kg as weak is retrospective. In receiver operating characteristic (ROC) analysis, the cut-off value is 25 kg for morbidity(Figure 6A) and 22 kg for mortality.(Figure 6B) Hand-grip strength less than 25 kg has a sensitivity of 72.41% and specificity of 84.37% to predict morbidity. Hand-grip strength less than 22 kg has a sensitivity of 75% and specificity of 79.25% to predict mortality.

In the analysis of hand-grip strength in the setting of esophageal cancer for elective radical esophagectomy with reconstruction, we found it to be a good predictor for outcome. We have reasons to suggest test for hand grip strength as a routine clinical tools for patients requiring elective esophagectomy with reconstruction. The results of the hand-grip strength did not indicate any specific physiology defect that other tests fail to detect. In contrast, the results of hand-grip strength can be viewed as a summary of overall physiological status [5,6].

The strength of hand-grip was also related to bone density [7]. In patients with femoral neck fracture, hand-grip strength was found to be a good indicator to predict the occurrence of both mortality and morbidity [8]. Whether the results can be applicable to other patient group is not known. However, it is worthy to evaluate its role in patients planed to undergo an major operation..

The limitation of the study is the small number of patients included in the study and follow-up duration is short.

## Conclusion

Patients with weak hand grip strength have higher risks of complication and mortality after elective esophagectomy with reconstruction. We recommend the test to be included in routine preoperative evaluation.

## Competing interests

The authors declare that they have no competing interests.

## Authors' contributions

CH Chen proposed the idea, designed the study and wrote the whole article.

H Chang participated in the design of the study and carried out the statistics.

YZ Huang and TT Hung collected patients' data and references. All authors read and approved the final manuscript.
